# Improving Outcomes Through Radiologic Verification: A Quality Improvement Approach to Nasogastric Tube Placement

**DOI:** 10.7759/cureus.81887

**Published:** 2025-04-08

**Authors:** Sonal Kumar, Yasmine White, Taylor Collignon, Edward Noguera

**Affiliations:** 1 Surgery, Ross University School of Medicine, Miramar, USA; 2 Anesthesiology, Cleveland Clinic Florida, Weston, USA; 3 Internal Medicine, Lake Erie College of Osteopathic Medicine, Bradenton, USA

**Keywords:** artificial intelligence, chest radiograph, critical care, deep learning, electromagnetic guidance, nasogastric tube

## Abstract

In critical care, nasogastric (NG) tube placement is a routine procedure and is necessary for enteral feeding, medication administration, and gastric decompression. Regardless, misplacing NG tubes continues to be a common issue and can result in severe complications, such as aspiration, pneumothorax, and gastrointestinal perforation. Although chest radiographs are the gold standard imaging test to verify the placement of an NG tube, misinterpretation is still a problem, particularly for non-radiologists. Our case report is a quality improvement initiative that explores the role of radiologic verification in improving outcomes and preventing complications associated with misplaced NG tubes. We present the case of a patient whose NG tube was misplaced in his lung. We also examine the limitations of radiographic imaging as the standard method for confirming NG tube placement. We examine the potential for improving patient safety by providing radiographic training, standardizing interpretation protocols, and integrating advanced technologies such as artificial intelligence (AI)-assisted detection. Our case report explores the importance of continuous quality improvement efforts in optimizing NG tube placement accuracy and reducing associated risks, ultimately enhancing patient care and safety.

## Introduction

Nasogastric (NG) tube placement is a routine procedure in critical care and is essential for enteral feeding, medication administration, and gastric decompression [1]. Regardless, misplacing NG tubes is still a recurring problem and leads to severe complications, such as aspiration, pneumothorax, and gastrointestinal perforation [2]. Chest radiographs are the standard imaging studies to confirm NG tube placement; however, misinterpretation remains a challenge, especially among non-radiologists [3].

In fact, studies have highlighted challenges in identifying NG tube positions on radiographs. There is even variability among diagnostic radiographers when interpreting NG tube placement on chest radiographs, suggesting the potential for errors even among trained professionals [4]. Additionally, placement complications such as tube migration into the gastric mucosa can further complicate interpretation and increase the risk of adverse events [5].

Advancements in artificial intelligence (AI) have introduced promising solutions for detecting malpositioned NG tubes. Studies showed the efficacy of deep learning tools in identifying misplaced tubes on portable supine chest X-rays, offering a potential adjunct to traditional radiographic evaluation [6,7-11]. Beyond radiographic methods, alternative technologies such as electromagnetic guidance have been explored to improve placement accuracy. Research showed that electromagnetic confirmation with chest radiographs is a viable method for verifying tube positioning in critical care settings [2].

Given the risks associated with misplaced NG tubes and the limitations of current confirmation techniques, there is an ongoing need for enhanced training, improved detection methods, and the integration of novel technologies to ensure patient safety. This report explores the accuracy of NG tube placement identification and emerging alternatives to radiographic confirmation.

## Case presentation

A 75-year-old male with a past medical history of heart failure with reduced ejection fraction (HFrEF), atrial fibrillation on anticoagulation, hyperlipidemia, hypertension, squamous cell carcinoma of the lung status post pneumonectomy with bowel metastases status post palliative radiation therapy to the left upper lobe mass, acute cholecystitis, and urothelial carcinoma of the bladder presented to the emergency department with complaints of weakness and anemia.

The patient reported using a pleural catheter every other day for drainage of a chronic pleural effusion. The patient was scheduled for an elective ventral hernia repair and a laparoscopic cholecystectomy, with the possibility of conversion to open. On admission, he denied fever, chills, nausea, vomiting, bowel pattern changes, melena, hematochezia, hematuria, or dysuria.

**Figure 1:**
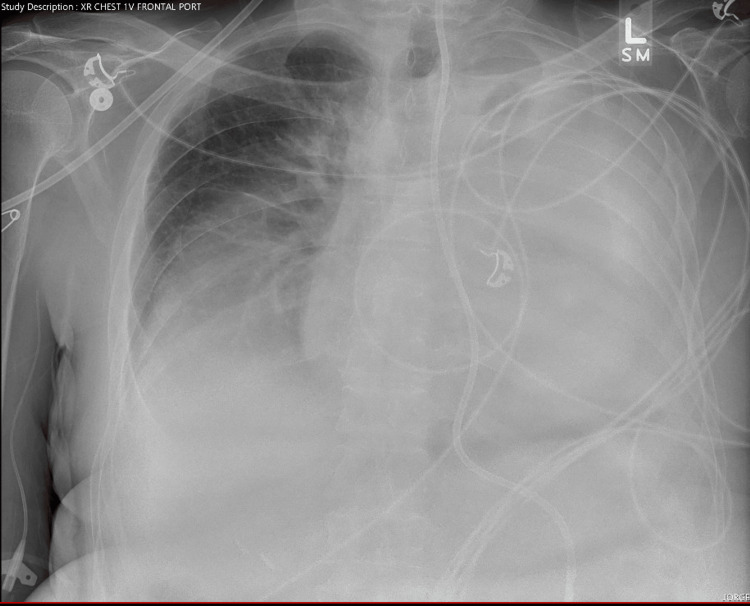
Chest radiograph obtained prior to nasogastric tube insertion.

The patient initially underwent laparoscopic cholecystectomy with indocyanine green (ICG) imaging. Twelve days later, the patient presented for an esophagogastroduodenoscopy (EGD) to facilitate percutaneous endoscopic gastrostomy (PEG) tube placement (Figure 1).

**Figure 2:**
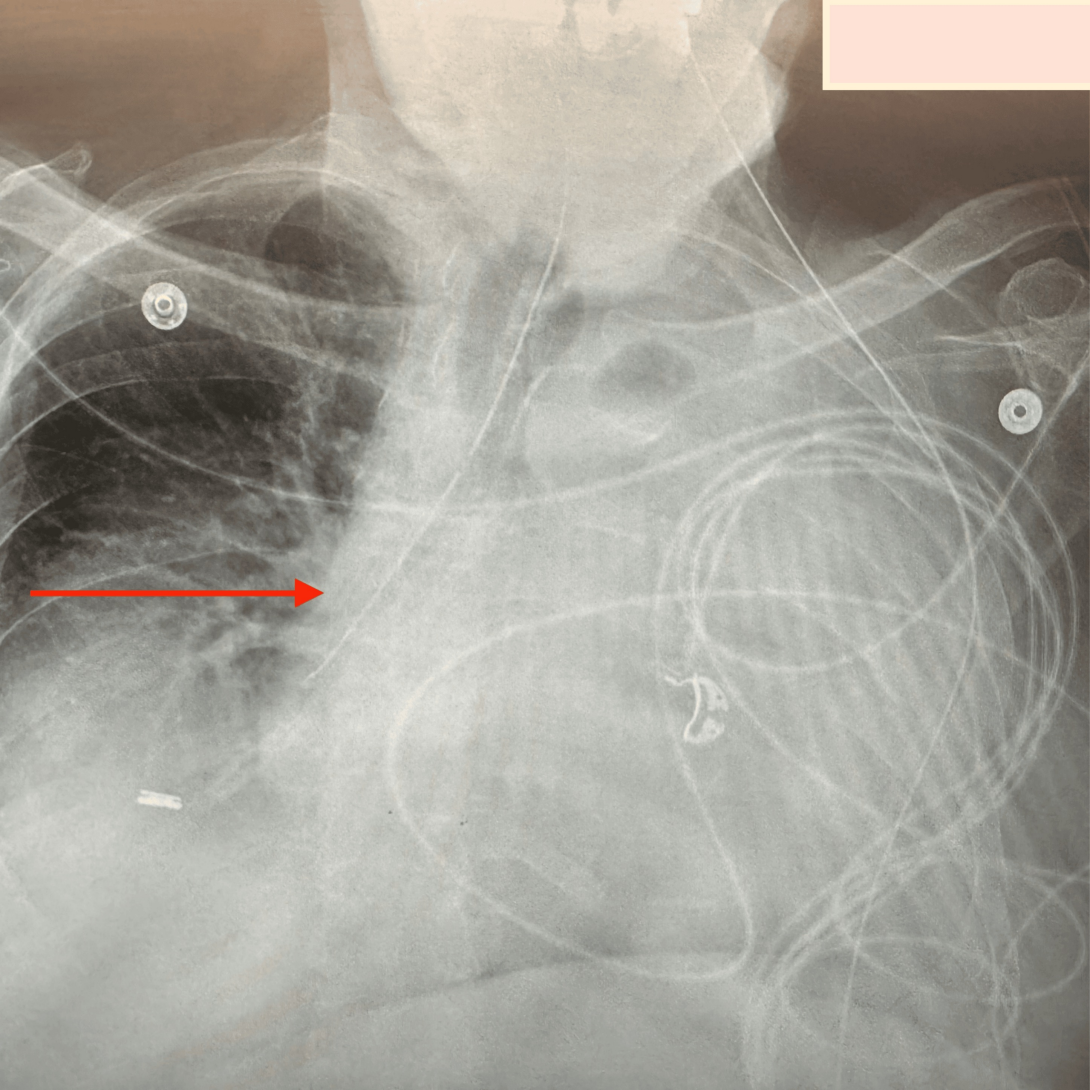
Chest X-ray showing a nasogastric (NG) tube mistakenly placed in the right lung (red arrow).

During the procedure, the Corflo feeding tube was exchanged, and a nasogastric (NG) tube was blindly inserted. The nurse subsequently contacted the surgical intern to request a chest X-ray to confirm proper tube placement, at which point it was found that the NG tube was misplaced (Figure 2).

Medications via this newly placed NG tube were given. Subsequently, the patient experienced a pulseless electrical activity (PEA) arrest, advanced cardiac life support (ACLS) protocol was initiated and the patient was intubated during resuscitation. Return of spontaneous circulation (ROSC) was achieved in about 15 minutes, with palpable carotid pulses. He was then transferred to the surgical intensive care unit (SICU) for post-cardiac arrest management, where he underwent targeted temperature management (TTM) with cooling and rewarming.

The patient ultimately stabilized hemodynamically but remained ventilator-dependent, necessitating tracheostomy placement, which he tolerated well.

## Discussion

We highlight the potential complications with NG tube placement, including the risk of placement in the tracheobronchial tree. NG tube insertion is a routine procedure. Traditional NG placement is done blindly, and bedside confirmation techniques include auscultation of the epigastric area to listen to air bubbles in the gastric chamber. However, this technique is operator dependent and can miss NG tubes placed in the airway.

In more difficult placements when the NG tube coils in the oropharynx, more invasive techniques under direct vision with a laryngoscope are an alternative; however, sedation may be required due to the invasive nature of the procedure. Errors in NG tubes can lead to severe, sometimes life-threatening, complications, such as aspiration, pneumothorax, and gastric or esophageal perforation [1]. These complications can be avoided, stressing the importance of using adequate placement techniques and prompt, accurate confirmation of NG tube placement.

Traditional confirmation methods primarily rely on chest radiographs, but misinterpretation of these images remains a significant concern. Studies have shown that the accuracy of NG tube position interpretation on chest radiographs can be highly variable, even among experienced diagnostic radiographers [2]. Even trained professionals are not immune to errors when interpreting radiographs [2]. This can lead to delayed recognition of malpositioned tubes, contributing to adverse events such as the one seen in this case.

Additionally, complications such as migration of the NG tube into the gastric mucosa, which may be difficult to detect on conventional radiographs, can exacerbate the challenges in managing NG tube placements [5]. Complications can lead to delayed diagnoses and worsen clinical outcomes, which is in line with the unfortunate progression of events in this patient [5]. These findings further emphasize the need for more accurate and reliable methods of confirming NG tube placement.

Recent advancements in artificial intelligence (AI) and machine learning have shown potential in improving the detection of malpositioned NG tubes. The efficacy of deep learning algorithms in detecting misplaced NG tubes on portable chest X-rays, which are commonly used in intensive care and emergency medicine settings [6]. Deep learning algorithms demonstrated high accuracy in detecting nasogastric tubes (NGTs) on neonatal radiographs, achieving an average precision (AP) of 0.977. This suggests that AI can reliably identify NGT placement and potentially reduce misplacement errors, offering a valuable tool for radiologists to enhance efficiency and patient safety [7]. These AI-based solutions have the potential to reduce human error, offering a promising adjunct to traditional radiographic methods. AI’s ability to identify misplacement with greater precision could lead to more timely interventions, potentially preventing complications like those seen in this case.

In addition to AI, alternative confirmation techniques, such as electromagnetic devices, have been explored to improve the accuracy of NG tube placement. The research compared the use of an electromagnetic device with chest radiography and found it to be a reliable method for confirming NG tube position in critically ill patients [3]. This approach provides real-time feedback on tube positioning, reducing the reliance on potentially inaccurate radiographs and offering a more direct confirmation method. Such technologies could prove valuable in clinical settings, particularly where frequent radiographic imaging may not be ideal.

Finally, the question of whether trainees can safely identify malpositioned NG tubes has also been raised. There are potential risks associated with relying on trainees or other non-radiology staff to interpret NG tube position, given the challenges in accurately identifying misplacements, especially in high-pressure clinical environments [4]. This highlights the need for comprehensive training for all healthcare providers involved in NG tube management, ensuring that they are equipped to handle potential complications effectively.

While chest radiographs remain the standard for confirming NG tube placement, their limitations in accuracy, especially among non-radiologists, necessitate the exploration of alternative confirmation methods. AI-assisted detection and electromagnetic guidance are promising tools that may improve the reliability of NG tube placement confirmation. As these technologies continue to evolve, it is crucial to integrate them into clinical practice to reduce errors, enhance patient safety, and prevent the severe complications associated with malpositioned NG tubes.

## Conclusions

Our case explores not only the accuracy of nasogastric tube placement in critical care but also the complications of misplacement, such as gastric perforation, respiratory distress, and aspiration pneumonitis. Even though there is widespread use of chest radiographs as the standard method for confirming NG tube position, variability in interpretation among healthcare providers remains a significant concern. As discussed in this example of a procedural complication requiring resuscitation and intensive care, accurate detection and prompt intervention are essential for patient safety. Advanced technology, such as artificial intelligence, offers promising solutions for improving the accuracy of NG tube placement confirmation and minimizing the risks associated with misplacement. Additionally, additional training for healthcare providers may improve patient safety and prevent adverse outcomes. Research that shows the adoption of these technologies in clinical practice could lead to a more reliable standard of care for NG tube confirmation. In the end, this may reduce complications and improve overall patient care.
